# Supplementary material for: Effect of interfaces on the nearby Brownian motion
Source: Nat Commun. 2015 Oct 6;6:8558. doi: 10.1038/ncomms9558 (PMC4600737; doi:10.1038/ncomms9558)
Supplement: Supplementary Information — Supplementary Figure 1, Supplementary Table 1, Supplementary Notes 1-2 and Supplementary References [file ncomms9558-s1.pdf]

Supplementary Figure

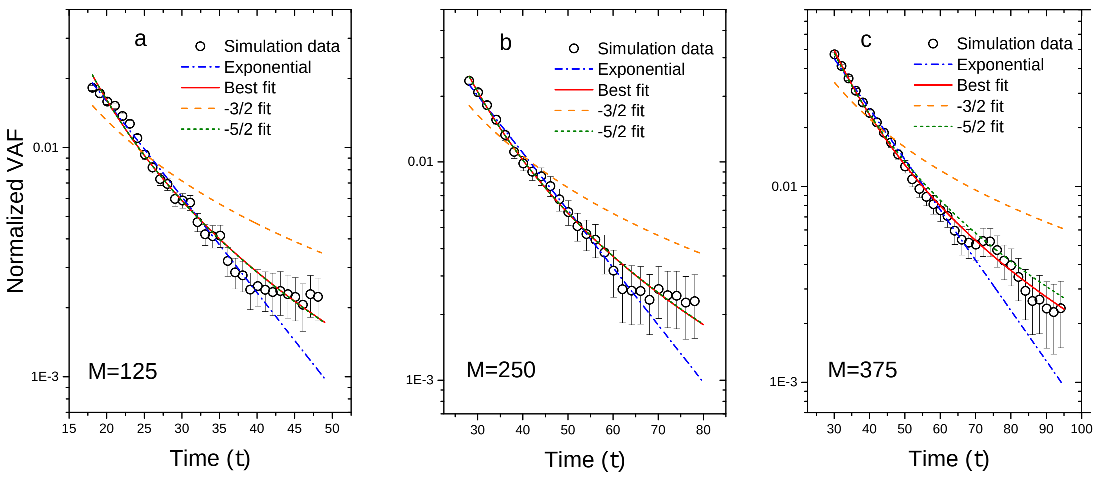

Supplementary Figure 1. Fittings of the asymptotic behavior of the near-boundary velocity autocorrelation functions (VAFs). The long-time tails of VAF are fitted to power law functions $y=ax^b$ (b=-3/2, b=-5/2 and b being a free fitting parameter) and to an exponential function. From the left to the right: the mass of the particle M=125, 250 and 375 in reduced units. See Supplementary Table 1 for the parameters of the fits.

Supplementary Table

Supplementary Table 1. Fitting results of the asymptotic behavior of the near-boundary VAFs. The long-time tails of VAF are fitted to power law functions $y=ax^b$ (b=-3/2, b=-5/2 and b being a free fitting parameter) and to an exponential function. R^2 represents the quality of the fit.

Mass	Fit with b=-5/2	Fit with b=-3/2	Best fit	Exponential
125	R^2=0.980	R^2=0.835	b=-2.49, R^2=0.980	R^2=0.976
250	R^2=0.996	R^2=0.849	b=-2.51, R^2=0.996	R^2=0.986
375	R^2=0.996	R^2=0.826	b=-2.64, R^2=0.996	R^2=0.990

Supplementary Notes

Supplementary Note 1. The Lorentz approximation.

In the main text we report a good agreement of the near-boundary diffusion constant calculated from our simulation and theoretically predicted by Lorentz[1]. It is worth noting that Lorentz's calculation is based on the assumption of $a=R/h\ll1$, where R is the hydrodynamic radius of the particle and h is the distance between the particle and the solid surface. Such calculation only deals with the first order perturbation from a on the diffusion constant. A more general theory taking into account higher order terms has been developed later by Faxén[2] who found that the near-boundary diffusion constant can be written as: $D_w=D_b(1\text{-}9/16a+1/8a^3\text{-}45/256a^4\text{-}1/16a^5+\ldots)$. Interestingly, there is no second order term in this equation and the third and fourth order terms have opposite signs, which means it is possible for them to cancel each other. Indeed, we found that in our case (where a=0.6) Faxén's exact solution with up to the fifth order term in a gives a result within 0.1% of the Lorentz approximation. Therefore the good agreement between our simulations and Lorentz theoretical prediction shown in Fig. 2 of the main text also holds for comparison with the more exact Faxén's theory.

Regarding the VAF of the Brownian particle, present theories including the one developed by Felderhof have not yet taken into account the higher order terms in a. Within the first order approximation, different theories predicted qualitatively different asymptotic behaviors $t^{-3/2}$ vs $t^{-5/2}$ long-time tail near a boundary). For example, in the theory of Felderhof[3], the leading term of the VAF is $A_w t^{-5/2}$ with the coefficient A_w being a function of a. Including higher order terms in a will not change the order in the leading term of time (the power of the long-time tail), but it could modify the coefficient A_w (the amplitude of the tail). Nevertheless, it is possible that the contribution from higher order terms of a is small, as a similar cancellation could happen as in the case of diffusion constant. Our simulation result for the heaviest nano-particle also suggests that the Lorentz approximation (with only first order term in a) works reasonably well in predicting the VAF of Brownian motion close to the boundary. For the lightest nano-particle, the discrepancy between theory and simulation is mainly due to insufficient time scale separation between the Brownian motion and the boundary relaxation, as discussed in the main text.

Supplementary Note 2. Relation between the long-time tail and hydrodynamic resonance.

The hydrodynamic long-time tail of a free-diffusing Brownian particle is the result of the backflow of fluid acting on the particle. Once a harmonic trap or confining potential is introduced, the coupling between the trap and the backflow can lead to a hydrodynamic resonance. Such a resonance is manifested as a peak in the power spectral density (PSD) of the trapped Brownian particle. The VAF and PSD functions are in time and frequency domain, respectively. These two quantities are connected by the Fourier transform of the drag coefficient or admittance of the Brownian motion[4]. Intuitively speaking, a more significant (of larger amplitude) long-time tail allows a stronger hydrodynamic resonance and vice versa. The experiment by Jannasch *et al*[5] revealed a suppressed hydrodynamic resonance near a boundary, which signifies a reduced long-time tail in VAF. This experimental observation was explained by the model of Berg-Sørensen and Flyvbjerg[6], which predicted a $t^{-3/2}$ long-time tail near a boundary but with a reduced amplitude. This explanation has been, however, debated. As discussed in the main text, the correct near-boundary long-time tail is $t^{-5/2}$ as predicted by Felderhof and other

theorists[3]. We have shown that the amplitude of such long-time can be reduced by the relaxation of boundary.

Supplementary References

1. Lorentz, H. A. *Abhandlungen über Theoretische Physik* (Teubner Verlag, 1907).
2. Faxén, H. Ark. Mat. Astron. Fys. **17**, 1 (1923).
3. Felderhof, B. U. Effect of the wall on the velocity autocorrelation function and long-time tail of Brownian motion. *J. Phys. Chem. B* **109,** 21406-21412, (2005).
4. Felderhof, B. U. Spectrum of position fluctuations of a Brownian particle bound in a harmonic trap near a plane wall. *J. Chem. Phys.* **136,** 144701, (2012).
5. Jannasch, A., Mahamdeh, M., and Schaeffer, E. Inertial effects of a small Brownian particle cause a colored power spectral density of thermal noise. *Phys. Rev. Lett.* **107,** 228301, (2011).
6. Berg-Sørensen, K. and Flyvbjerg, H. Power spectrum analysis for optical tweezers. *Rev. Sci. Instrum.* **75,** 594-612, (2004).
